# Castleman Disease Presenting as a Mediastinal Mass

**DOI:** 10.7759/cureus.18409

**Published:** 2021-09-30

**Authors:** Sebastian Garcia, Jerry Fan

**Affiliations:** 1 Internal Medicine, Baylor Scott & White Medical Center, Temple, USA

**Keywords:** castleman disease, lymphoproliferative disorder, mediastinal mass, unicentric, multicentric

## Abstract

Castleman disease is a complex benign lymphoproliferative disorder characterized by the enlargement of a single lymph node or a group of lymph nodes. Its etiology is unclear, with the mechanism of action of IL-6 and HHV-8 implicated as possibly associated with the development of the disease. Diagnosis depends on the histopathological findings of the involved lymph nodes. Surgical resection can be curative, but a small number of cases may be unresectable and need radiation and chemotherapy with subsequent resection if possible.

## Introduction

Castleman disease (CD) is a rare lymphoproliferative disorder characterized by the involvement of lymph nodes [1]. CD can be classified on the basis of the involvement of a single lymph node (or a single region of lymph nodes) (unicentric CD (UCD)) versus multiple regions of lymph nodes (multicentric CD (MCD)) [2]. CD can be difficult to diagnose as histology and pathology from the biopsy are not unique to CD but can also be present in other disease processes. We present a case of a previously healthy male who developed a large mediastinal mass consistent with CD that did not respond to chemotherapy or radiation.

## Case presentation

**Figure 1:**
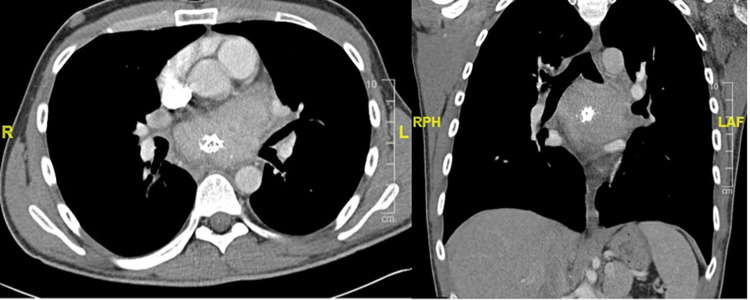
Chest CT demonstrating large mediastinal mass measuring 8.2 cm. Left: axial Right: coronal

A 25-year-old male with a history of type 1 diabetes mellitus presented to the hospital with a two-month history of shortness of breath, wheezing, chest tightness, and palpitations, which was treated with albuterol and prednisone without significant improvement. He subsequently developed odynophagia, night sweats, and occasional diarrhea, and he lost 20 lb of weight. He underwent a chest computed tomography (CT), which revealed a large mediastinal mass measuring 8.2 cm within the subcarinal region with internal calcification, compression, and narrowing of the right main pulmonary artery and left mainstem bronchus (Figure 1).

**Figure 2:**
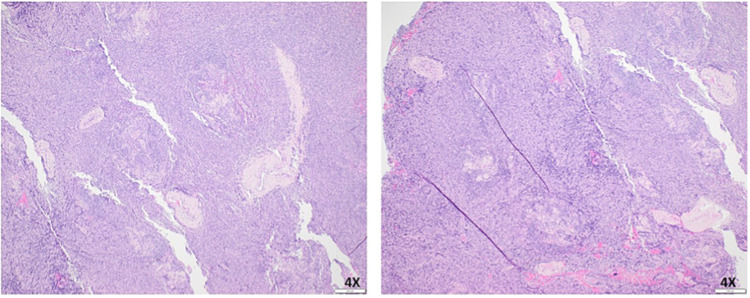
Histological sections of the subcarinal mass specimen showing features of Castleman disease. The lesion has a prominent follicular component composed of hyalinized germinal centers surrounded by linear arrays of mantle zone cells (the so-called “onion skin”). In addition, there is a prominent stromal component between these follicles that is composed of many small lymphocytes associated with increased blood vessels and fibrosis.

Endobronchial ultrasound with biopsy and cervical mediastinoscopy were performed, but cytology was inadequate for diagnostic purposes. A video-assisted thoracoscopic surgery biopsy with adequate tissue revealed prominent follicular components composed of hyalinized germinal centers surrounded by linear arrays of mantle zone cells (onion skin). Prominent stromal components between those follicles, which are composed of many small lymphocytes associated with increased blood vessels and fibrosis, were also noted. These features were characteristic of a hyaline vascular variant of Castleman disease. Flow cytometry was nondiagnostic with mixed T- and B-lymphocytes with a polyclonal B-lymphoid predominance (Figure 2).

Unfortunately, due to significant vascular involvement and adherence to critical structures, the mass was deemed to be unresectable. He underwent chemotherapy with rituximab and then cyclophosphamide, doxorubicin, vincristine, and prednisone (CHOP) with radiation but had minimal response.

## Discussion

Castleman disease (CD) is a rare lymphoproliferative disorder distinguished by two distinct forms: unicentric and multicentric [1]. Unicentric CD (UCD) involves a single lymph node or a single region of lymph nodes, while multicentric CD (MCD) involves multiple areas of enlarged lymph nodes [1]. UCD is often asymptomatic unless the mass compresses critical structures, causing secondary symptoms [2]. UCD is more common in the mediastinum but can occur in lymph nodes from any area of the body, including the axilla, neck, retroperitoneum, or abdomen [3].

The pathogenesis of UCD remains unclear, but viral, inflammatory, and neoplastic mechanisms have been proposed by investigators to be associated with the development of the disease [1]. IL-6 dysregulation has been proposed to be the mechanism of the development of CD [4]. IL-6 is involved in the differentiation of lymphocytes, production of acute-phase reactants, angiogenesis, and tumorigenesis [4]. Patients with CD can have elevated levels of IL-6 that normalize with the removal of the involved lymph nodes [4]. HHV-8 has also been implicated in the development of CD [5].

CD can be difficult to diagnose. IL-6 levels and the presence of human immunodeficiency virus should be evaluated as dysregulation of IL-6 and susceptibility of human herpesvirus-8 in those with human immunodeficiency virus are implicated in the development of CD [6]. Biopsy is the gold standard for the diagnosis of CD, and microscopic examination helps differentiate UCD “hyaline vascular variant” from MCD “plasma cell variant” [6,7].

Treatment for CD mainly involves surgical removal of the mass, which is usually curative [8]. Unfortunately, some patients may experience compression or adhesion of critical vessels or organs, as was evident in our patient, making surgical resection impossible. In these circumstances, radiation therapy could be used as adjuvant therapy in hopes of shrinking the tumor prior to surgical resection [8-10]. Our patient underwent radiation therapy and treatment with rituximab and subsequently CHOP. Unfortunately, he had minimal response to therapy and is currently in a long-term acute care hospital recovering from respiratory failure.

## Conclusions

Castleman disease is often difficult to diagnosis as it remains asymptomatic until a significant mass effect causes secondary symptoms. A tissue biopsy is essential to the diagnosis of Castleman disease.

## References

[1] Fajgenbaum DC, Shilling D (2018). Castleman disease pathogenesis. Hematol Oncol Clin North Am.

[2] Mitsos S, Stamatopoulos A, Patrini D, George RS, Lawrence DR, Panagiotopoulos N (2018). The role of surgical resection in unicentric Castleman's disease: a systematic review. Adv Respir Med.

[3] Talat N, Belgaumkar AP, Schulte KM (2012). Surgery in Castleman's disease: a systematic review of 404 published cases. Ann Surg.

[4] El-Osta HE, Kurzrock R (2011). Castleman's disease: from basic mechanisms to molecular therapeutics. Oncologist.

[5] Wu D, Lim MS, Jaffe ES (2018). Pathology of Castleman disease. Hematol Oncol Clin North Am.

[6] Abramson JS (2019). Diagnosis and management of Castleman disease. J Natl Compr Canc Netw.

[7] Wojtyś M, Piekarska A, Kunc M (2019). Clinicopathological comparison and therapeutic approach to Castleman disease-a case-based review. J Thorac Dis.

[8] Hountis P, Dedeilias P, Douzinas M (2008). The management of Castleman's disease of the mediastinum: a case report. Cases J.

[9] Haap M, Wiefels J, Horger M, Hoyer A, Müssig K (2018). Clinical, laboratory and imaging findings in Castleman's disease - the subtype decides. Blood Rev.

[10] Uysal B, Demiral S, Gamsiz H, Dincoglan F, Sager O, Beyzadeoglu M (2015). Castleman's disease and radiotherapy: a single center experience. J Cancer Res Ther.

